# Mapping patterns of metastatic lymph nodes for postoperative radiotherapy in thoracic esophageal squamous cell carcinoma: a recommendation for clinical target volume definition

**DOI:** 10.1186/s12885-019-6065-7

**Published:** 2019-09-18

**Authors:** Jing Yu, Wen Ouyang, Chunyang Li, Jiuling Shen, Yu Xu, Junhong Zhang, Conghua Xie

**Affiliations:** 1 Department of Radiation and Medical Oncology, Zhongnan Hospital of Wuhan University, Wuhan, Hubei People’s Republic of China 430071; 2 Hubei Key Laboratory of Tumor Biological Behaviors, Zhongnan Hospital of Wuhan University, Wuhan, 430071 Hubei China; 3 Hubei Cancer Clinical Study Center, Zhongnan Hospital of Wuhan University, Wuhan, 430071 Hubei China

**Keywords:** Mapping patterns, Recurrent nodes, Postoperative radiotherapy, Thoracic esophageal squamous cell carcinoma

## Abstract

**Background:**

The clinical target volume (CTV) for postoperative radiotherapy for thoracic esophageal squamous cell carcinoma (TESCC) needs to be defined. The study aim was to map metastatic lymph nodes (LNMs) in a computed tomography (CT)-based atlas and delineate the postoperative radiotherapy target area.

**Methods:**

Sixty-nine TESCC patients with first recurrent regional LNMs after esophagectomy were included. The LNM epicenters were registered onto corresponding anatomic axial CT images of a standard patient in the treatment position, with reference to the surrounding vascular and bony structures. The LNM sites were based on lymph node map of esophageal cancer, AJCC 8th. The lymph metastasis risk for different segments of thoracic esophagus was assessed.

**Results:**

One hundred and seventy-nine LNMs were mapped onto standard axial CT images. The upper-middle mediastinum region (station 1 to 8 M) contained 97% of metastases in the upper segment of thoracic esophagus, 90% in the middle segment, and 66% in the lower one. Advanced pathological stage (≥IIIB) might be a predictive factor for upper abdominal region (UAR) relapse in lower TESCC. Lower cervical para-tracheal LNMs were within a 4.3-cm bilaterally expanded area from the midline of the body and a 2.2-cm expanded area from the anterior of vertebral body, from the superior border of the C7, to the inferior border of the first thoracic vertebra.

**Conclusion:**

A modified target from the upper border of C7 to the lower border of caudal margin of the inferior pulmonary vein level could cover the high-risk area of TESCC underwent postoperative radiotherapy. UAR seems to be an elective irradiation target for lower TESCC at pathological IIIB stage and higher.

**Electronic supplementary material:**

The online version of this article (10.1186/s12885-019-6065-7) contains supplementary material, which is available to authorized users.

## Background

Esophageal squamous cell carcinoma is the most common type of cancer, and also the fourth leading cause of cancer-related death in China [1]. Overall, more than half of the tumors are primarily located in thoracic segments and are in stage II and III at the time of presentation [2]. Based on the landmark CROSS study, preoperative chemoradiation followed by surgery is the preferred treatment strategy for resectable stage II and III thoracic esophageal carcinoma [3]. However, since the relative low proportion of squamous cell carcinoma enrollment, it is difficult to extrapolate the conclusions to both squamous cell carcinoma and adenocarcinoma. A recent study from china confirmed the potential value of preoperative chemoradiation for thoracic esophageal squamous cell carcinoma (TESCC), though a longer follow-up result was still needed [4].

Since lack of prospective evidence, postoperative radiotherapy has not determined as a standard initial care for locally advanced TESCC. However, a large population-based study found that esophagectomy in the real-world was not a rare initial treatment for thoracic esophageal carcinoma. A total of 4893 patients were chosen to receive initial esophagectomy (including adenocarcinoma and squamous cell carcinoma) between 2008 and 2011 the United States [5]. We also found there were 2422 TESCC cases underwent initial esophagectomy alone from 2004 to 2014 in Surveillance Epidemiology and End Results database [6]. Postoperative radiotherapy could improve the outcomes for pathological T_3/4_, N_0/+_ TESCC [5–8]. Based on these findings, postoperative radiotherapy seems to be an important adjuvant therapy for TESCC.

Until now, the clinical target volume (CTV) of postoperative radiotherapy for TESCC has not been established, even though several previous studies have tried to explore the postoperative pattern of relapse for defining the target [9, 10]. The results showed supraclavicular and superior mediastinal lymph nodes areas might have the high risk of relapse. However, there is nearly not enough evidence of the involved sites on computed tomography (CT) images.

Therefore, the aim of this study was to map the locations of the first recurrent lymph nodes (LNMs) in CT images and to guide CTV delineation in postoperative radiotherapy for TESCC.

## Methods

### Patients

Between September 2011 and September 2018, a total of 214 TESCC were treated with esophagectomy but without adjuvant or neo-adjuvant radiation therapy in our institution. Among them, 121 patients occurred local regional recurrence. Of all recurrent cases, 69 patients could provide the first recurrent lymph nodes location in their diagnostic axial CT images. The recurrent lymph node needed to be re-evaluated by two experienced radiological experts. Tumor-positive nodes on radiographic images were identified by the following characteristics: when lymph node showing round shape with a short axis length ≥ 1 cm, the presence of an infiltrative margin, an increase in number and size compared with previous CT images, the presence of central necrosis or non-homogeneous enhancement, and responsiveness to treatment.

### Metastatic lymph node delineation

The locations of the first recurrent lymph nodes were defined based on the lymph node map of esophageal cancer published in the 8th edition of AJCC [11]. Accordingly, regional lymph node stations 1 to 8 M were defined as the upper-middle mediastinum region (UMMR); stations 8Lo, 9, and 15 were defined as the inferior mediastinum region (IMR); and stations 16 to 20 were defined as the upper abdominal lymph node region (UAR).

One patient without mediastinal lymph node metastasis who underwent total marrow irradiation therapy at our center was selected as a standard patient. She was simulated in the supine position with arms at the sides of her body. Two radiation oncologists and a radiologist worked together to transfer the LNM locations onto the corresponding anatomic positions in the axial CT images of the standard patient, with reference to the surrounding vascular and bony structures. All nodes were plotted with a diameter of 6 mm. If a node was larger than 6 mm, the geometric center was contoured as their center with a diameter of 6 mm. With regard to the lower cervical para-tracheal nodes (station 1 region), their positions in relation to the vertebrae, and the distance of each to the midline of the body was recorded. The LNMs were plotted in the Varian planning system (version 13.5; Varian Medical Systems, Palo Alto, CA, USA).

### Statistical analysis and image processing

Categorical and continuous variables were analyzed using SPSS version 20.0 (IBM Corp., Armonk, NY, USA). Image post-processing was carried out using the MIM image analysis platform (MIM software, Cleveland, OH).

## Results

### Patient characteristics

In our cohort, the median time to disease recurrent was 9 months (range, 5–47 months) and the median overall survival was 15 months (range, 11–47 months). The majority of patients were at pathological stage II (*n* = 19, 27.5%) and III (*n* = 30, 43.5%). Primary middle TESCC was found in the majority of patients. The most common surgical procedure was Ivor-Lewis (*n* = 41, 59.4%); it was followed by the McKeown (*n* = 15, 21.7%) and Sweet procedures (*n* = 13, 18.9%). Adjuvant chemotherapy was administered only in 17 cases (24.6%). Table 1 lists the patient characteristics.

**Table 1:** Patient characteristics

Variable	Value
Age (y), median (range)	56 (44–80)
Sex	
Male	60 (87.0%)
Female	9 (13.0%)
Surgical type	
McKeown	15 (21.7%)
Ivor-Lewis	41 (59.4%)
Sweet	13 (18.9%)
Location	
Upper	11 (15.9%)
Middle	31 (44.9%)
Low	27 (39.2%)
Grade	
Well	8 (11.6%)
Median	32 (46.4%)
Poor	24 (34.8%)
Undifferentiated	5 (7.2%)
Stage	
I	12 (17.4%)
II	19 (27.5%)
III	30 (43.5%)
IVa	8 (11.6%)
Adjuvant chemotherapy	
Yes	17 (24.6%)
No	52 (75.4%)

### Recurrence lymph nodes distribution

**Fig. 1:**
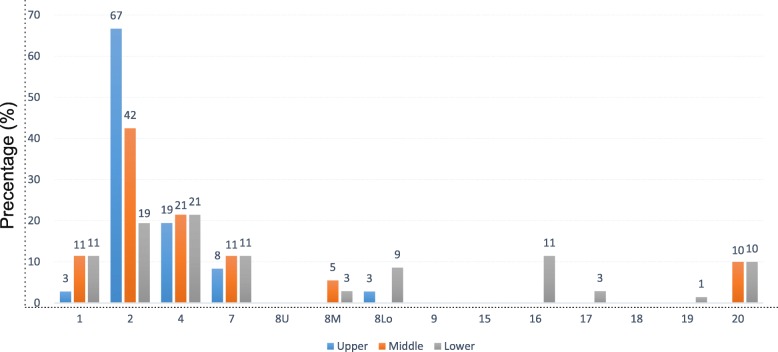
Frequency of lymph node recurrence according to the lymph node map for esophageal cancer found in the 8th edition of the AJCC. AJCC, American Joint Committee on Cancer. Blue column, upper thoracic esophageal squamous cell carcinoma; Orange column, middle thoracic esophageal squamous cell carcinoma; Gray column, lower thoracic esophageal squamous cell carcinoma

**Fig. 2:**
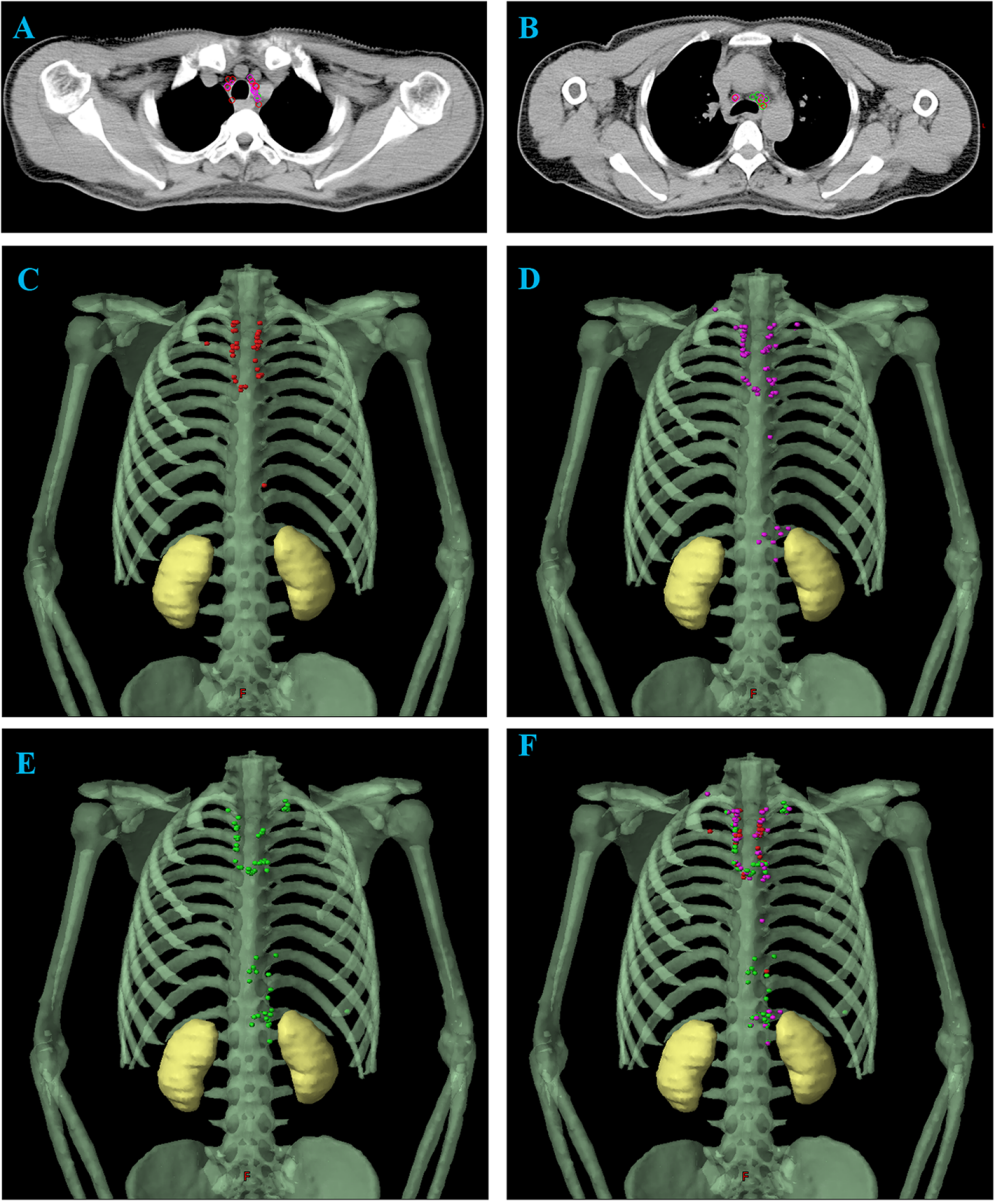
LNM map for TESCC. **a**. Upper paratracheal node area; **b**. Lower paratracheal node area; **c**. LNM map of the upper thoracic segment; **d**. LNM map of the middle thoracic segment; E. LNM map of the lower thoracic segment; **f**. LNM map of all the thoracic segments. LNM, metastatic lymph node; TESCC, thoracic esophageal squamous cell carcinoma; Red plot, metastatic lymph nodes of upper thoracic esophageal squamous cell carcinoma; Purple plot, metastatic lymph nodes of middle thoracic esophageal squamous cell carcinoma; Green plot, metastatic lymph nodes of lower thoracic esophageal squamous cell carcinoma

A total of one hundred and seventy-nine LNMs were mapped onto the standardized axial CT images. As shown in Fig. 1, UMMR was the most common site of LNMs for all segments of thoracic esophagus. In the UMMR, the paratracheal node regions (station 2/4) showed the highest percentage of lymph node involvement, accounting for 38.0% of metastasis in station 2 and 20.7% of metastasis in station 4 respectively (Fig. 2a & b). In addition, the UAR, which included stations 16 to 20, showed a medium risk of lymph node involvement, as it contained 14.0% (25/179) of the total LNMs and 25.7% (18/70) in lower segment. Finally, the IMR exhibited only a slight risk of lymph node involvement (3.9% of the total LNMs).

### Recurrence lymph nodes map in different thoracic segments

Next, we investigated the metastatic risk in different thoracic segments. The recurrence map showed that upper and middle TESCC had a similar relapse pattern (Fig. 2c & d). UMMR was still the area with the highest recurrence, as it showed 97 and 90% recurrence in the upper and middle segments respectively. Lower TESCC showed a more extensive distribution of LNMs. Even though a moderate metastatic risk was observed in the UAR (25.7%), the UMMR was sill the dominant site of relapse (66.0%) (Fig. 2e & f).

### Recurrence lymph nodes in lower cervical Para-tracheal region

**Fig. 3:**
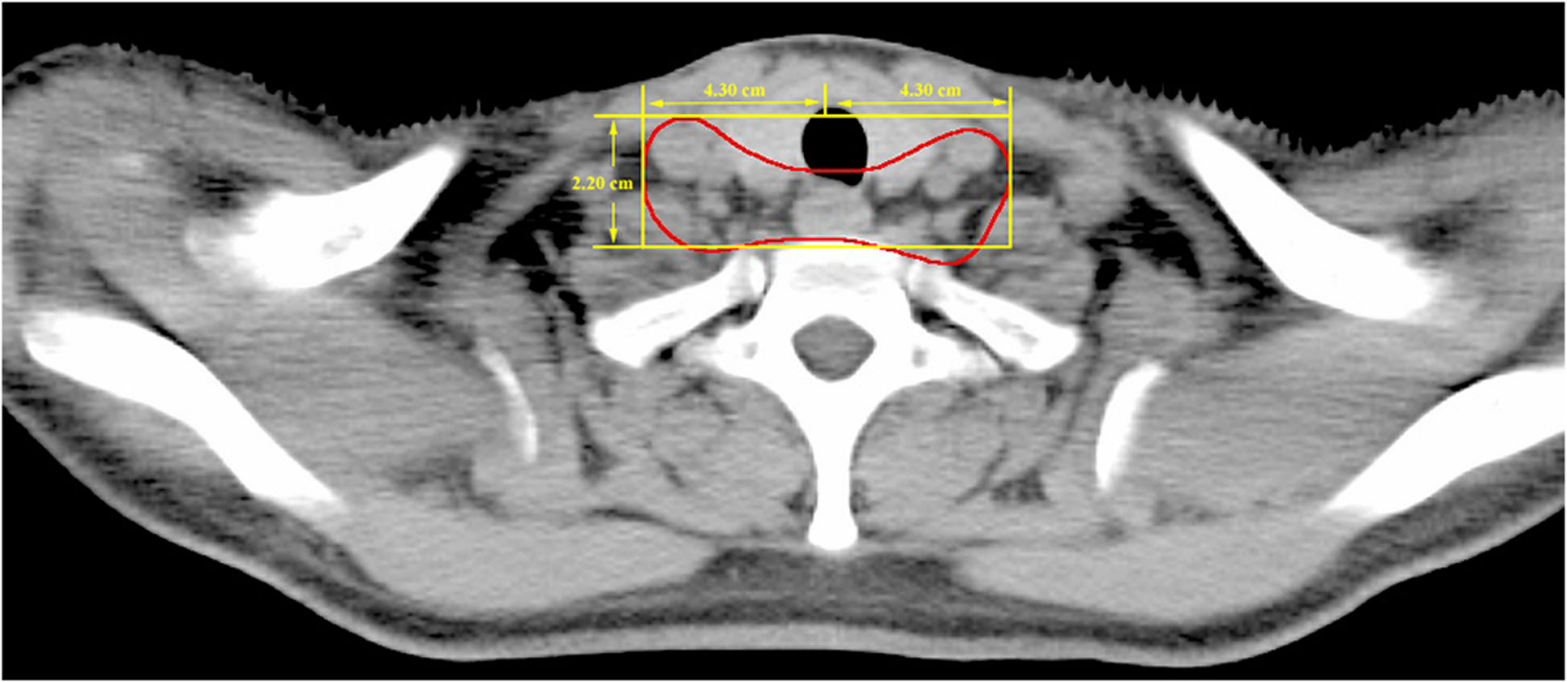
Recurrent region in the lower cervical para-tracheal region

A total of 17 LNMs showed relapse in the lower cervical para-tracheal region (station 1). They were all within a region that included a 4.3-cm area that spread bilaterally from the midline of the body and a 2.2-cm area that spread from the anterior border of the vertebral body, from the superior border of the 7th cervical vertebra (C7), to the inferior border of the first thoracic vertebra (Fig. 3). This indicates that a relatively small prophylactic irradiation target for covering the lower cervical para-tracheal region might be adequate.

## Discussion

According to the latest LNM maps of esophageal cancer and the results in this study, we found that the UMMR had the highest risk of recurrence for upper and middle TESCC. For lower TESCC, although a medium risk was found in the UAR, the UMMR still had a higher probability of nodal involvement. Therefore, a modified target from the upper border of C7 to.

The lower border of caudal margin of the inferior pulmonary vein level could be sufficient to cover most of the high-risk recurrence area of all TESCC after surgery; this target area carries a recurrence risk of 97% for upper TESCC, 90% for middle TESCC and 66% for lower TESCC. With regard to lower TESCC, in patients at a more advanced pathological stage (≥IIIB), the UAR could be added as an elective postoperative irradiation target.

The CTV for TESCC has been investigated by several pioneer studies. In one such study based on the locoregional recurrence data from 160 patients who underwent radical surgery, Liu concluded that the supraclavicular and superior mediastinal areas should be a routine radiation target for TESCC [12]. Cai and colleagues reported similar results in another study population [9]. However, their results did not provided a specific delineation of CTV on CT images. In our CT-based LNM map, we found that the UMMR had high metastasis risk for all segments TESCC. Therefore, this area should be included in CTV. Since the use of precise radiotherapy techniques is on the increase, our CT-based imaging results may provide more practical clinical guidance. Another retrospective study that specifically analyzed the recurrence pattern of the lower TESCC showed that the UAR had the first recurrence percentage of 12%, but it still reported a higher metastatic risk for the UMMR [13]. However, we observed a higher recurrence probability of 25.7% in UAR area, which was comparable to the report by Cai [9]. A total of 7 lower TESCC patients developed first recurrence in the UAR in our study. After reviewing the clinical characteristics of this subpopulation, we found that most of them (6/7) were in pathological stage IIIB or above (Additional file 1). Accordingly, we speculated that primary lower TESCC coupled with a more advanced pathological stage might elevate metastatic risk in the UAR. Considering the bimodal recurrent risk of lower TESCC, we particularly recommended UAR as an alternative postoperative ENI for lower TESCC with stage IIIB and higher.

Definitive chemoradiation was an appropriate option for patients who decline surgical therapy or those with unresectable cases. Until now, irradiation target of the definitive chemoradiation has not well established. Some research considered the involved-field irradiation could provide similar outcomes with less treatment related toxicities than elective nodal irradiation (ENI) for cervical and upper or middle TESCC [14, 15]. We found with the increase of the inclusion proportion of lower disease, patients would benefit more from ENI [16, 17]. Therefore, using additional ENI might also improve the outcomes of lower TESCC underwent definitive chemoradiation. On account of recent metastatic map, we supposed UAR might also serve as an optional ENI for lower TESCC undergoing definitive chemoradiation.

Studies have reported that McKeown esophagogastrectomy (with three incisions) is appropriate for the middle and lower third thoracic esophageal cancer. The greater range of lymphadenectomy with this procedure might decrease locoregional recurrence and improve overall survival [18, 19]. As shown in Additional file 2, we did not observe any significant decline in metastatic risk in the paratracheal node regions (station 2/4) in cases in which the McKeown procedure was performed. It is possible that radical dissection in the superior mediastinal areas elevates the risk of injury in recurrent laryngeal nerves. A compromised dissection in this region might fail to improve locoregional control rate (LCR) in the UMMR. Despite this, a predominant advantage of this surgery would be improvement in the LCR in the lower cervical para-tracheal region (station 1), the metastatic risk was lower in this region than that in the previous report [12].

The delineation of the lower cervical para-tracheal region (supraclavicular region) of TESCC is somewhat ambiguous. Two studies recommended that the high-risk region extended from the lower border of the cricoid cartilage to the upper edges of the clavicle heads, with the lateral boundary extending to the medial border of the carotid sheath [20, 21]. Our CT-based data validated a similar supraclavicular irradiation target in terms of the lateral and anterior border, but our mapping showed a smaller irradiation target in the craniocaudal plane. A modified irradiation area in lower cervical para-tracheal region would decrease the radiation-induced toxicities in supraclavicular region such as radiothermitis or cutaneous erythema.

The main limitations of our study are that it was based on data from a single institution and that the sample size was small. Another limitation could be that the location of the primary tumors was quite heterogeneous. To overcome this, we analyzed the pattern of relapse in different thoracic esophagus segments. Previous studies that have defined CTV were largely based on the result of lymph node dissection [10, 16, 17]. Recently, the target recommendation based on the mapping of metastatic nodes was found to be more reasonable and understandable [22, 23]. Because of the strict eligibility criteria, the sample sizes in these studies were rather moderate. Therefore, the clinical significance of the optimized CTV treatment should also be evaluated in the perspective clinical trials for TESCC patients underwent postoperative radiotherapy or chemoradiotherapy.

## Conclusion

Based on the pattern of recurrent nodal metastasis, we defined an atlas for post-operative radiotherapy clinical target volume that includes the cranial boundary of C7 to the caudal boundary of the inferior pulmonary vein. Additionally, UAR should be considered as an elective target volume for lower TESCC at pathological stage IIIB and higher.

## Additional files

Additional file 1: Clinical characteristics of lower TESCC patients with UAR recurrence. The clinical characteristics of lower upper thoracic esophageal squamous cell carcinoma patients with upper abdominal region recurrence. (DOC 36 kb)

Additional file 2: Frequency of lymph node recurrence in patients underwent McKeown surgery. Frequency of lymph node recurrence in patients underwent McKeown procedure according to the lymph node map for esophageal cancer found in the 8th edition of the AJCC. (PDF 382 kb)

## Data Availability

The datasets used in the current study available from the corresponding author on reasonable request.

## Abbreviations

AJCC: American Joint Committee on Cancer
C7: 7th cervical vertebra
CT: Computed tomography
CTV: Clinical target volume
IMR: Inferior mediastinum region
LCR: Locoregional control rate
LNMs: Metastatic lymph nodes
TESCC: Thoracic esophageal squamous cell carcinoma
UAR: Upper abdominal region
UMMR: Upper-middle mediastinum region
