# Supplementary material for: Mapping patterns of metastatic lymph nodes for postoperative radiotherapy in thoracic esophageal squamous cell carcinoma: a recommendation for clinical target volume definition
Source: BMC Cancer. 2019 Sep 18;19:927. doi: 10.1186/s12885-019-6065-7 (PMC6749673; doi:10.1186/s12885-019-6065-7)
Supplement: Supplementary file 1 — Clinical characteristics of lower TESCC patients with UAR recurrence. The clinical characteristics of lower upper thoracic esophageal squamous cell carcinoma patients with upper abdominal region recurrence. (DOC 36 kb) [file 12885_2019_6065_MOESM1_ESM.doc]

| Additional file 1. Clinical characteristics of lower TESCC patients with UAR recurrence | | | | | |
| --- | --- | --- | --- | --- | --- |
| Num. | Sex | Surgical  Type | pTNM  stage | Differentiation | Pathological  stage |
| 1 | Male | Sweet | T_2_N_2_M_0_ | G2 | IIIB |
| 2 | Male | Ivor-Lewis | T_3_N_2_M_0_ | G2 | IIIB |
| 3 | Male | Ivor-Lewis | T_4_N_2_M_0_ | G3 | IVA |
| 4 | Male | McKeown | T_3_N_2_M_0_ | Gx | IIIB |
| 5 | Male | Ivor-Lewis | T_4a_N_0_M_0_ | G2 | IIIB |
| 6 | Male | McKeown | T_4a_N_1_M_0_ | G3 | IIIB |
| 7 | Male | Sweet | T_3_N_1_M_0_ | G3 | IIIA |

TESCC, Thoracic esophagus squamous cell carcinoma; UAR, Upper abdominal lymph node region.
