# Supplementary figures and images for: Mapping patterns of metastatic lymph nodes for postoperative radiotherapy in thoracic esophageal squamous cell carcinoma: a recommendation for clinical target volume definition
Source: BMC Cancer. 2019 Sep 18;19:927. doi: 10.1186/s12885-019-6065-7 (PMC6749673; doi:10.1186/s12885-019-6065-7)

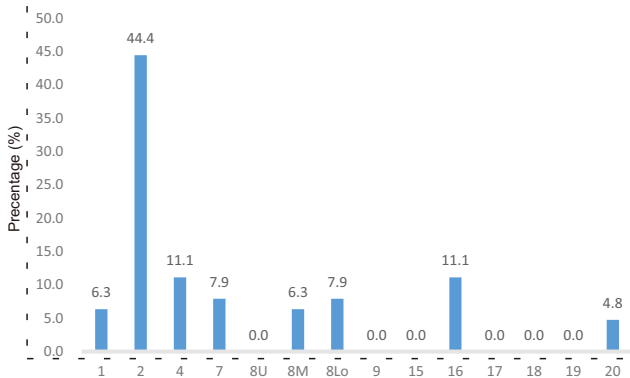

Supplement: Supplementary file 2 — Frequency of lymph node recurrence in patients underwent McKeown surgery. Frequency of lymph node recurrence in patients underwent McKeown procedure according to the lymph node map for esophageal cancer found in the 8th edition of the AJCC. (PDF 382 kb) [file 12885_2019_6065_MOESM2_ESM.pdf]
